# The London Hospital Saturday Fund and the National Insurance Act

**Published:** 1913-02-22

**Authors:** 


### THE LONDON HOSPITAL SATURDAY FUND AND THE NATIONAL INSURANCE ACT.

### Some Views and Fears of the Saturday Fund Executive.

The year 1912 will be remembered as an anxious one in the hospital world. Institutions and associations which support them were alike perplexed as to the changes in their management which will become necessary when the provisions of the National Insurance Act, 1911, become fully operative. The effect of the National Insurance Act upon workmen's contributions and also those of their employers were indeed serious on the income of this Fund in the second half of 1912. For in many cases the latter supplement the contributions of the former and in not a few encourage the collections by giving as much as their workpeople. During the first half of the year the income of the Fund continued to increase, as it has done without intermission since the discontinuance of the annual street collection in 1897, and at Midsummer Day was £600 in advance of that for the corresponding period of 1911. Had this rate been maintained the receipts for the year would have been £3,000 more than they were. Unfortunately, however, a considerable number of collectors sent back their penny-a-week collecting sheets at Michaelmas with the pennies scored for July 6 and 13, but not for subsequent Saturdays. The payments by employers and employed under the Act came into force on July 15. After this date contributions collected by means of boxes and annual sheets also showed a shrinkage, as did likewise the special collections.

### A Vigorous Effort.

Many thousands of circulars, posters, and pamphlets, explanatory of the work of the Fund and of the Act were distributed over Greater London. Never since the Fund was established had the knowledge of its work been more widely disseminated or its benefits more bountifully granted. Never had a philanthropic institution, whose interests were threatened by a new Act of Parliament, worked more assiduously and loyally than this Fund in making clear the intentions and purposes of the National Insurance Act, 1911. Owing to the splendid manner in which the honorary workers responded to the call, the sum of £10,431 was forwarded to the Head Office between December 30, 1912, and January 13, 1913, when the accounts for 1912 were closed. This brought up the total for the year to £35,088. A further sum of £10,041 was received by the Distribution, Surgical Appliance, and Ambulance Committees in part payment of the cost of benefits supplied; so that the grand total for the year was £45,129.

It will be seen that, notwithstanding the perplexity caused by the operations of the National Insurance Act in the last half of 1912, the General Fund for that year was only £1,844 less than that for 1911. This was mainly due to the hearty co-operation of the local committees and the honorary collectors at contributing firms.

| Receipts | 1910 | 1911 | 1912 |
|---|---|---|---|
| | £ | £ | £ |
| General Fund ... ... ... | 34,694 | 36,932 | 35.088 |
| Distribution Committee ... | 6 250 | 7,013 | 8.347 |
| Surgical Appliance Committee. | 1,276 | 1,443 | 1,621 |
| Ambulance Committee ... ... | 91 | 80 | 73 |
| Totals ... ... ... | £42,311 | £45 468 | £45.129 |
| Number of Benefits Granted ... | 59,737 | 63,361 | 65,267 |

### Prospects of the Saturday Fund.

The prospects of the Hospital Saturday Fund are decidedly promising for two reasons. First, there is no secular institution in this or any other country with a larger number of zealous voluntary workers than that possessed by this Fund. Week in and week out, year in and year out, the honorary collectors and members of standing and local committees, numbering at least 11,000, loyally perform their self-imposed duties. Further, there are many benefits obtainable from this Fund which are not promised under the Act. The more important of these are the Ambulance, Convalescent Home, Dental, and Surgical Appliance Benefits. Moreover, all benefits are intended for subscribers, their wives, and children. The objects of the Fund are twofold: It aims not only at supporting the voluntary hospitals, but also at helping its supporters who are in need. In 1912 it made grants amounting to £32,306 to hospitals and kindred institutions, and granted 65,267 benefits to its supporters. In many respects it will complete and supplement the work done by the National Insurance Act.

### Effects of National Insurance on Hospital Admissions.

The effects of the National Insurance Act upon the ready admission of the artisan class to the hospitals immediately they require hospital care compared with what it was formerly in London cannot be given at present. Most of the large hospitals have adopted what are known as the St. Bartholomew's resolutions. Some such regulations, with modifications to meet varying conditions, were doubtlessly necessary in the changed circumstances. These new regulations have been operative for a few weeks only at the time of writing. Consequently, as regards in-patient treatment, no opinion at present can be expressed. But, with regard to out-patient treatment, especially casualties, no withholding of the facts would be justified. From the day that the medical benefits under the Act became available there has been from all parts of Greater London a constant stream of complaints from the honorary collectors of the Fund. Out-patients have been refused further treatment, minor casualties, such as simple fractures, incised or lacerated wounds, foreign bodies in the eye, doubtful injuries to the chest or abdomen, and the like, have been sent away from many hospitals without first treatment, or even examination, to be attended to by some distant, unknown, or unavailable Insurance doctor. It is common knowledge that in London the panels of doctors are incomplete and that thousands of the industrial classes do not know the names and addresses of their Insurance doctors. In the provinces the panels are more or less complete, and insured persons can more readily find their appointed doctors. Moreover, in London no days of grace have been granted to insured persons to realise the changed conditions and to arrange accordingly; whilst in the provinces three, or even six, months are wisely being allowed for making known and preparing for the new procedure. No wonder then that the outcry among the industrial classes of London has been so marked and so general; no wonder that indignation has been aroused and ill-feeling engendered! It has taken forty years to induce the industrial classes of London to give systematic support to the voluntary hospitals; it may not take more than forty weeks to alienate their support. We do not believe that the hasty and harsh action complained of has been done with the knowledge and approval of the managing bodies of the voluntary hospitals; but the harm has been done, and we urge the responsible bodies to see that their new regulations are carried out in a kindly, liberal spirit, so that, before it is too late, the goodwill of the working classes may not be lost. We ask that insured persons requiring hospital care, as understood prior to January 15, should be allowed some days of grace; that they should be examined by a doctor; that they should have first treatment, if necessary; and that injured persons, even though they may not be serious cases, working many miles away from home, may receive treatment for the sake of humanity, and in order, it may be, to save a day's pay.

The Future of Metropolitan Hospitals and Saturday Fund.

It is difficult to say what the future may have in store for the Metropolitan hospitals and such funds as the Hospital Saturday Fund. There is a want of unity, of co-operation, of co-ordination in the work of the voluntary hospitals—a want which might be met by a Central Hospital Board, or such association as the British Hospitals Association, on which all the hospitals should be fairly represented. Then a common policy, especially as regards insured persons, might be adopted and followed. New regulations are necessary. Insured persons, not as a matter of charity but of right, should be entitled to consultative advice and special medical and surgical treatment at hospitals. This can be most economically done through the voluntary hospitals, which, however, should be paid *pro ratâ* for the advice, treatment, and accommodation provided. But it should be done in such a way as not to impair the voluntary hospital system. It must be remembered that the voluntary hospitals do not now exist for the benefit of the necessitous poor only. The Poor-Law infirmaries provide for the poorest classes. The voluntary hospitals accommodate all others who require expert advice and treatment and cannot afford to pay for them. But they also accommodate a considerable proportion of the community who can afford to pay moderate fees and who are referred to them by general practitioners for professional or other reasons. Furthermore, they are training grounds for the medical and nursing professions, and in them the doctors and surgeons who attend to the rich have access to the most expensive apparatus, opportunities for studying interesting cases, for keeping themselves up to date in medical and surgical science, and for acquiring and retaining the skill so necessary for performing their professional duties to the rich. It goes without saying that the managing bodies of the voluntary hospitals are representatives of the subscribers, past and present. May it not be justly said that, apart from the benefactions of our ancestors, the hospitals are supported, it may be inadequately, by the voluntary contributions, not of one class only, but of all classes? Can anyone affirm that the industrial classes do nothing in support of them? In London, besides many contributions given directly to individual hospitals, some give through King Edward's Hospital Fund, the League of Mercy, the Hospital Sunday Fund, the Hospital Saturday Fund, and many other sources. In the provinces the voluntary hospitals are supported by the industrial classes to a larger extent than in London, because it is an easier matter to organise the collections. As matters stand at present we fear that the future of the voluntary hospitals in London, and all collecting agencies on their behalf, will be very uncertain; nay, more, that the voluntary system in the Metropolis may receive such a blow as will be deplored by all sections of the community.

